# SG-APSIC1109: Redesign of outpatient clinic clean care to decrease postoperative SSIs after orthopedic implants

**DOI:** 10.1017/ash.2023.93

**Published:** 2023-03-16

**Authors:** Andaru Dahesihdewi, Tri Hartatik, Susi Wijayanti, Ryantinah, Andaru Dahesihdewi

**Affiliations:** Sardjito Hospital, Yogyakarta, Indonesia; Sardjito Hospital, Yogyakarta, Indonesia; Sardjito Hospital, Yogyakarta, Indonesia; Sardjito Hospital, Yogyakarta, Indonesia; Sardjito Hospital, Yogyakarta, Indonesia

## Abstract

**Objectives:** Based on Sardjito Hospital surveillance data in 2020, the incidence of SSI in orthopedic implant surgery was 46 cases (4.7%), mostly in the outpatient clinic. We evaluated some of the potential risks and proposed redesign of infection prevention and control measures in April 2021 to improve the overall clean care at the orthopedic outpatient clinic. **Methods:** We conducted an operational study to redesign various components of clean care using a before-and-after evaluation of infection risk. The study was led by an IPC nurse and was supported by all levels of stakeholders at Sardjito Hospital, a referral and academic hospital in Yogyakarta, Indonesia, during May–September 2021. **Results:** The redesigned components covered continuing professional development (CPD) through a workshop on clean care and wound care for doctors and nurses. The workshop also encouraged high-level management to make several important changes: (1) to redistribute medical staff schedules, (2) to start online patient registration to better distribute and decrease patient loads, (3) to set up the waiting room as well as the dressing room with strictly separate between dirty and clean areas, (4) to schedule daily general disinfection at noon during service hours, and (5) to perform routine air disinfection after daily clinic services as well as placing an additional portable HEPA filter for continuous air disinfection. After the these changes, during 2021, 7 SSIs occurred among postoperative orthopedic implant patients, a decrease of 85%. We observed more clean and neat rooms without patient overcrowding as well as easy and comfortable flow of patients and staff. Environmental pathogen germ counts decreased significantly. **Conclusions:** A redesign project at the orthopedic outpatient clinic reduced the incidence of postoperative SSIs and reduced the number of environmental pathogens. Overall clean care is a basic strategy in IPC for improving patient safety.

